# Nanoparticles in influenza subunit vaccine development: Immunogenicity enhancement

**DOI:** 10.1111/irv.12697

**Published:** 2019-11-27

**Authors:** Atin Khalaj‐Hedayati, Caroline Lin Lin Chua, Peter Smooker, Khai Wooi Lee

**Affiliations:** ^1^ School of Biosciences Faculty of Health and Medical Sciences Taylor's University Subang Jaya Malaysia; ^2^ Department of Biosciences and Food Technology School of Science RMIT University Bundoora Victoria Australia

**Keywords:** immunogenicity, influenza vaccine, nanoparticles, subunit vaccine, vaccine delivery

## Abstract

The threat of novel influenza infections has sparked research efforts to develop subunit vaccines that can induce a more broadly protective immunity by targeting selected regions of the virus. In general, subunit vaccines are safer but may be less immunogenic than whole cell inactivated or live attenuated vaccines. Hence, novel adjuvants that boost immunogenicity are increasingly needed as we move toward the era of modern vaccines. In addition, targeting, delivery, and display of the selected antigens on the surface of professional antigen‐presenting cells are also important in vaccine design and development. The use of nanosized particles can be one of the strategies to enhance immunogenicity as they can be efficiently recognized by antigen‐presenting cells. They can act as both immunopotentiators and delivery system for the selected antigens. This review will discuss on the applications, advantages, limitations, and types of nanoparticles (NPs) used in the preparation of influenza subunit vaccine candidates to enhance humoral and cellular immune responses.

## 1 INTRODUCTION

Influenza virus infection is a global public health problem, causing a huge morbidity and mortality burden due to annual epidemics and pandemics. Worldwide, annual epidemics cause 3 to 5 million cases of severe illness, and about 290,000 to 650,000 deaths.^1^ Vaccination is the most effective method to prevent influenza infection. Current influenza vaccines mainly rely on hemagglutinin (HA) proteins as antigens to induce neutralizing antibodies that can inhibit virus infection and replication in humans. These antibodies are mostly targeting the immunodominant epitopes of the influenza virus that are highly variable between different virus strains. Each year, new variants of influenza virus may emerge due to antigenic drift, which necessitates the reformulation of influenza vaccines on a yearly basis.^2^ Previously, mismatches between predicted and actual circulating strain have resulted in reduced vaccine protection and increased clinical cases.^3^ A “universal” vaccine that targets the conserved regions of influenza viruses and induces a broadly protective immunity may dramatically improve protection against seasonal and pandemic influenza viruses. Antibodies that target conserved sites in the HA stalk have been isolated from humans and shown to confer protection in animals challenged with various influenza virus strains and subtypes.^4^ However, it is noteworthy that antibodies specifically targeting the conserved HA2 region can also increase disease severity by enhancing viral fusion to target cells, hence should be given sufficient consideration during universal vaccine design and evaluation.^5^

Licensed influenza vaccines are currently available as inactivated (whole inactivated virus vaccine, split virus vaccine or subunit vaccine), live attenuated, and recombinant vaccines (Table 1). These vaccines are produced in eggs or cell cultures and mainly induce antibodies against strain‐specific HA.^6^ Today, research is more focused on the development of subunit vaccines, as they are safer and easier to produce. With recombinant technology, the production of epitopes of interest, such as the conserved stalk domain of HA, can be done. In addition to vaccine antigens, adjuvants are also sometimes added to vaccines to boost immunogenicity. Adjuvants are particularly important in the development of influenza vaccines for the elderly population who has decreased immune capacity and during pandemics, where a rapid antibody response is required.^7^ In addition, adjuvants are also required in the development of novel peptide‐based influenza vaccines, which are known to have low immunogenicity. There are currently six types of adjuvants that have been included in licensed influenza vaccines; alum, AS03, AF03, MF59, heat labile enterotoxin, and virosome, which is a nanoparticle (NP).

**Table 1:** Currently available influenza vaccines for the 2019‐2020 influenza season in the United States^112^

Vaccines	Manufacturers	Production	Preparation	Type	Adjuvant
Afluria Quadrivalent	Seqirus Pty. Ltd.	Eggs	Inactivated	Split virus	‐
Fluad	Seqirus, Inc	Eggs	Inactivated	Purified subunit	MF59®
Fluarix Quadrivalent	GlaxoSmithKline Biologicals	Eggs	Inactivated	Split virus	‐
Flublok Quadrivalent	Protein Sciences Corporation	Insect cells	Recombinant	Subunit	‐
Flucelvax Quadrivalent	Seqirus, Inc	MDCK cells	Inactivated	Purified subunit	‐
FluLaval Quadrivalent	ID Biomedical Corporation of Quebec	Eggs	Inactivated	Split virus	‐
FluMist Quadrivalent	MedImmune, LLC	Eggs	Attenuated	Live virus	‐
Fluzone High Dose	Sanofi Pasteur, Inc	Eggs	Inactivated	Split virus	‐
Fluzone Quadrivalent	Sanofi Pasteur, Inc	Eggs	Inactivated	Split virus	‐

Recently, the potential use of NPs as adjuvants in vaccines has been gaining interest. The inclusion of NPs in vaccine formulations has been reported to enhance antigen stability,^8^, ^9^, ^10^ promote targeted antigen delivery,^11^, ^12^ and assist slow release of antigens 13, 14 to eliminate the need for booster shots.^15^, ^16^ Different NPs have been evaluated for their ability to deliver antigens and increase immune responses against influenza antigens in vaccines. The current review focuses on the latest scientific advancement in the application of different NPs in influenza subunit vaccine development to enhance immunogenicity (Table 2).

**Table 2:** Current development of nanoparticle‐based influenza vaccines

NPs	Vaccine candidate composition	Humoral response	Cellular response	Cross‐protection	Protective against lethal challenge	Animal species	Clinical phase	Reference
Spore	CotB‐M2e3 (H1, H2, H3)	√	√	N/A	√	Mice	Pre‐clinical	24
	B‐S‐HA	√	√	N/A	N/A	Chicken	Pre‐clinical	113
VLP	BV VLP‐HA‐NA‐M1	√	√	N/A	√	Mice	Pre‐clinical	38
	BV VLP‐HA‐NA‐M1	√	√	N/A	N/A	Mice and ferrets	Pre‐clinical	39
	HBc VLP‐M2e‐HA2 (Tandiflu1)	√	N/A	√	√	Mice	Pre‐clinical	43
	HBc VLP‐M2e‐NP	√	√	√	√	Mice	Pre‐clinical	44
	Influenza VLP‐HA (H1, H8, H13, H3, H4, H10)	√	N/A	√	√	Mice	Pre‐clinical	114
	Recombinant A (H1N1) 2009 influenza VLP vaccine (HA)	√	N/A	N/A	N/A	Human	Phase II	46
	gH1‐Qbeta (HA1)	√	N/A	N/A	N/A	Human	Phase I	61
	A(H7N9) VLP Antigen (HA, NA, M1)	√	N/A	N/A	N/A	Human	Phase I	47
	Plant‐based QVLP (HA)	√	√	√	N/A	Human	Phase I & II	115, 116
Phage VLP	T7‐M2e	√	√	√	√	Mice	Pre‐clinical	58
	QB‐M2e	√	N/A	N/A	√	Mice	Pre‐clinical	60
Polysaccharide	Chitosan‐DNA	√	√	√	√	Mice	Pre‐clinical	8
Liposome	HA/DC‐Chol:DPPC liposomes	√	N/A	N/A	N/A	Mice	Pre‐clinical	117
	Vaxfectin (TIV)	√	√	N/A	N/A	Mice	Pre‐clinical	78
Virosome	Inflexal V HA‐NA	√	√	N/A		Human	FDA‐approved	87, 118
ISCOM	Viral protein	√	√	√	√	Mice	Phase I	97
Gold	Au‐HA	√	√	N/A	√	Mice	Pre‐clinical	104

## 2 NANOPARTICLES IN INFLUENZA VACCINE DEVELOPMENT

NPs are comparable to pathogens in terms of their size (1‐1000 nm), and thus, they can be efficiently recognized by immune cells and can therefore act as carriers to induce desirable immune‐activating effects. NPs can facilitate the delivery of loaded antigens to the primary antigen‐presenting cells (APCs).^17^, ^18^ For efficient protection against influenza, influenza vaccines are required to induce specific antibody responses, such as antibodies belonging primarily to the immunoglobulin G (IgG) subclass that can block the function of HA, either via blocking host receptor binding or preventing fusion.^19^, ^20^ To promote vaccine immunogenicity, various types of NPs have been employed in the design of influenza subunit vaccines such as bacterial spores, virus‐like particles (VLPs), bacteriophages, polysaccharides, liposomes, virosomes, immune‐stimulating complexes (ISCOM), and inorganic NPs, which are reviewed here under “Natural” and “Synthetic” nanoparticle categories.

### 2.1 Natural nanoparticles

#### 2.1.1 Bacterial spore

Spores are quiescent cells that can be produced by certain bacterial species such as the Gram‐positive *Bacilli* and *Clostridia*.^21^ Spore formation is a survival strategy that enables the bacteria to survive in harsh environmental conditions. Typically, mature spores are 800‐1200 nm in size and have either a spherical or ellipsoidal shape.^22^ Interestingly, a spore can self‐assemble into its functional structure and when used as a vaccine carrier, it can protect the antigens on its surface from degradation and stimulate an immune response.^23^, ^24^ The spores of *B subtilis* have high stability, low production cost, facile construction, and a good safety profile which earns them the Generally Recognized as Safe (GRAS) status.^25^ Moreover, they can be administered via the oral pathway, where they can protect the antigens from degradation by stomach acid prior to reaching immune cells within the small intestine.^26^ In vaccine design, these bacterial spores are usually conjugated to vaccine antigens through recombinant technology. However, for them to work efficiently, the vaccine antigens need to be of certain size and complexity to effectively activate antigen presentation.^27^ Apart from that, the possibilities of transferring selectable marker genes, and release of live recombinant bacterial spores, are major concerns. Recently, antigen co‐administration and antigen adsorption to non‐recombinant spores were reported as safer alternatives.^28^, ^29^

*Bacillus subtilis* spores have been used in the design of an oral influenza vaccine, where the spore coat protein of *B subtilis* PY79 (CotB) was fused with three copies of conserved matrix protein (M2e). M2e is the ectodomain of M2 protein, a proton channel of the influenza virus, that is highly conserved across all human influenza virus A strains, thus making it one of the main targets for universal influenza vaccine development. The authors reported that M2e was successfully displayed on the spore surface and the recombinant spore (RSM2e3) exhibited significant immunogenicity in mice. Repeated immunization was shown to elicit M2e‐specific IgG (titer of 1:12,800 at week 17 post‐1st immunization), as well as strong cellular immune responses. When immunized mice were further challenged with A/PR/8/34 (H1N1) influenza virus, lung specimens revealed significantly lower levels of the virus titers compared to the control group, in addition to a 100% survival rate.^24^ In a similar study, a tandem repeat of four consensus sequences coding for the human‹avian‹swine‹human M2e (M2eH‐A‐S‐H) peptide was fused to *B subtilis* spore coat proteins and stably expressed on the spore surface. Oral immunization of mice with the recombinant spore carrying M2eH‐A‐S‐H was reported to elicit specific antibody production in the absence of any other adjuvant. However, the levels of antibody titers were relatively low, suggesting that the induced immunity was inadequate for protection and some modifications in vaccine preparation may be required to increase immunogenicity.^30^

Live and heat‐inactivated spores of *B subtilis* can also be directly used in vaccine production due to their ability in binding to influenza virions. In a previous study, mice that were intranasally immunized with killed spores adsorbed to H5N1 virions (NIBRG‐14) were fully protected even after being challenged with a lethal dose of the virus (˃20 times LD_50_). Particularly interesting was the observation that in the absence of influenza antigens, the killed spores alone were able to confer about 60% partial protection in the animals, suggesting that the spores themselves are immunogenic in nature.^31^ This type of protection however was short‐lived and has been attributed to the recruitment of natural killer cells into lungs in response to the killed spores.

#### 2.1.2 Virus‐like particles

Virus‐like particles (VLPs) are self‐assembling and non‐replicating particles that are devoid of infectious genetic material.^32^ VLPs can be produced from different host cells, which include bacteria, yeast, insect, and animal cell lines. They can be used as both particulate carriers and immunopotentiators in vaccine development due to their immunogenic characteristics such as having similar size to original pathogen, repetitive surface geometry, and ability to induce innate and adaptive immune responses.^33^ The main advantage of VLP‐based vaccines is that the immune system of the host can recognize VLPs in a similar way to the original virus to promote a robust immune response.^34^ They have been primarily designed to promote B‐cell activation and induce potent antibody responses following activation of T helper cells.^35^, ^36^

There are several licensed human prophylactic VLP‐based vaccines such as Cervarix®, Gardasil®, and Gardasil9® against human papillomavirus (HPV) and the third generation Sci‐B‐Vac™ vaccine against hepatitis B virus (HBV). VLP‐based approaches are also explored as a promising approach for the development of a universal influenza vaccine.^37^ To design a successful VLP‐based vaccine, the most applicable VLP construct has to be selected and antigens need to be incorporated without destabilizing the VLPs. To achieve this, each biological virus‐derived particle needs to be studied in detail for their properties and possible side effects before use in human.

Virus‐like particle‐based vaccines have been widely explored in the design of influenza vaccines, with promising results in providing protection against the infection. An influenza VLP‐based vaccine candidate consisting of influenza HA, NA, and matrix protein (M1) (H7N9 A/Shanghai/2/2013) was reported to successfully elicit strong humoral and cellular immune responses in mice when administered either via the intramuscular (IM) or intranasal immunization routes. Notably, only 10 μg of H7N9 VLPs was required to achieve a 100% survival rate against a lethal dose of H7N9 virus.^38^ Another study reported that influenza H3N2‐VLPs expressing HA, NA, and M1 proteins induced protective antibody responses with higher efficacy and potency than the whole inactivated vaccines in mice and ferrets.^39^ M2e5x is another VLP‐based vaccine candidate that has been genetically engineered to contain a tandem repeat of five M2e variants from human, swine, and avian influenza A viruses. It was shown to protect infected mice against a lethal challenge from distinct influenza A viruses (H3N2 and H5N1).^40^, ^41^ Additionally, M2e5x was able to increase the immunogenicity of split vaccines and promote cross‐protection when tested in ferrets.^42^

Tandiflu1 is an influenza VLP‐based vaccine candidate comprising of a hepatitis B virus core (HBc) VLPs fused to four conserved antigens from M2e and HA stalk. Vaccination with Tandiflu1 led to the production of cross‐reactive and protective antibodies, which resulted in 100% protection from a lethal influenza challenge with H1N1 in mice. In addition, serum transfer from vaccinated animals successfully conferred protection from influenza‐associated illness in naïve mice.^43^ A previous study which also similarly used HBc VLPs as carriers for three M2e protein and nucleoprotein (NP) epitopes was shown to induce potent humoral and cell‐mediated immunity in mice.^44^ A single vaccine shot that contains multiple VLPs against different virus subtypes can result in broad protection. Mice vaccinated with a mixture of influenza VLPs containing 4 different HA subtypes of influenza A viruses (H1, H3, H5, and H7) were protected from lethal challenges with same subtypes and also other hetero sub‐typic strains that were not included in the vaccine.^45^

A few VLP influenza vaccines have been tested in humans with promising results. For example, VLP vaccines against influenza A/California/04/2009 (H1N1) was tested in adults and showed good safety and immunogenicity profiles, where 82%‐92% of individuals who received a single dose of vaccine achieved ≥40 hemagglutinin inhibition (HAI) titer.^46^ For the recombinant VLP influenza A (H7N9) vaccine, the presence of ISCOMATRIX™ adjuvant was needed to induce protective antibodies. In the phase I clinical trial, 80.6% of subjects receiving adjuvanted H7N9 VLP vaccine developed HAI responses compared to 15.6% in the group who received a higher dose of non‐adjuvanted vaccine.^47^ Nanoflu, which is a quadrivalent VLP vaccine with Matrix‐M adjuvant, was tested in older adults (≥65 years old) at phase 2 clinical trial and reported to induce significant HAI responses.^48^, ^49^ Recently, the Quadrivalent VLP Influenza Vaccine comprising of H1, H3, and two B hemagglutinin proteins is being tested in elderly adults at phase 3 clinical trial.^50^

Interestingly, VLPs can be modified via recombinant technology to contain additional adjuvant, such as bacterial proteins to further enhance immunogenicity. Wang et al studied the effect of adding a modified *Salmonella* flagellin protein to influenza VLPs associated with HA and M1 proteins. They reported that these chimeric VLPs induced higher IgG2a and IgG2b levels and cytokine responses when compared to control VLPs without the flagellin protein. The chimeric VLPs not only conferred full protection against the homologous PR8 virus strain, but it also showed significant cross‐protection by having a 67% survival rate when challenged with a lethal dose of heterosubtypic H3N2 strain.^34^ Results from a clinical study showed that the HA influenza‐flagellin recombinant vaccine (VAX125) was highly immunogenic in a group of elderly population (≥65 years old).^48^ Another influenza vaccine, STF2.4 × M2e (VAX102), which consists of *Salmonella typhimurium* flagellin type 2 protein fused to M2e protein was tested in healthy, young individuals and reported to be safe and immunogenic at low doses (0.3‐1.0 μg).^51^ However, at higher doses (3 and 10 μg), the vaccine was associated with severe symptoms due to increased levels of C‐reactive protein.

#### 2.1.3 Bacteriophage VLPs

Phage VLPs are not pathogenic, and there is no pre‐existing immunity against them in humans; consequently, they are safer than other VLPs.^52^ Barfoot et al showed that T4 phages can be taken up by DCs as efficiently as the influenza virus.^53^ Bacteriophage VLP systems employ phage capsid proteins to display peptides or proteins on the surface of the phage. The cargo size is usually dependent on the types of phage. For example, bacteriophage T7 capsid proteins 10A and 10B can accommodate about 400 copies of peptide or protein with 50‐1200 amino acids. Gene VIII protein of Ff phage possesses higher display valency of up to 8000 copies on its filamentous body; however, only peptides and small proteins can be displayed. The gene III protein of Ff phage with low copy number tolerates larger displays, more effectively.^54^, ^55^, ^56^

Several phages have been developed as antigen adjuvant for influenza vaccine development. A previous study incorporated influenza conserved nucleoprotein (NP) into bacteriophage P22 through genetic engineering and immunized mice were able to generate anti‐NP antibodies and CD8+ T‐cell responses specific to NP. The vaccine candidate protected mice against both H1N1 and H3N2 influenza strains that were administered via intranasal challenge.^57^ Two previous studies, which displayed M2e peptide on T7 and f88 phages, demonstrated that the vaccine candidate elicited protective immune responses after three subcutaneous (SC) immunizations, where a high survival rate was observed after lethal challenge with H1N1 and H2N3 virus.^58^, ^59^ Cross‐linking of M2e peptide to the T7 phage VLPs is essential in promoting this protective response, as M2e peptides that were simply mixed with T7 VLPs did not elicit protection against disease.^58^ Another study, which genetically fused M2e to bacteriophage Qβ coat protein VLPs, induced a high level of M2e‐specific IgG and IgA antibodies in mice and completely protected the mice against a lethal challenge with the influenza virus PR8.^60^ An example of bacteriophage VLPs that have been used in human clinical trial is the gH1‐Qbeta vaccine against A/California/07/2009 (H1N1) which employs the use of RNA bacteriophage Qbeta. It was reported to induce higher antibody production compared to the same vaccine that was adjuvanted with alhydrogel, and comparable immunogenicity and safety profile to commercial vaccines.^61^ However, an important consideration in the design of these vaccines in the future is the possibility of developing anti‐phage antibodies in immunized individuals, hence impacting their overall balance of gut microbiota and health.^62^, ^63^

#### 2.1.4 Polysaccharide

Polysaccharides have various characteristics that make them suitable to be used for nanovaccine preparation, including having immunomodulatory effects and a good safety profile, as well as being biocompatible and biodegradable. They are natural polymers with glycosidically linked carbohydrate monomers.^64^, ^65^ Polysaccharides commonly comprise of chitosan and its derivatives and can function as vaccine adjuvants, given their ability to activate the immune system and promote antigen‐specific immune responses.^66^, ^67^, ^68^ Chitosan NPs have been previously used in influenza DNA vaccines and shown to exhibit high stability and high encapsulation rate. It was reported that the chitosan NP‐encapsulated DNA vaccine induced prolonged release of the plasmid DNA and effective immune responses are induced compared with DNA vaccine alone.^8^ Chitosan is approved by the US FDA for use in pharmaceuticals and food, but generally they have a few disadvantages such as poor solubility and low transfection rate that need to be considered before being used in influenza subunit vaccine development.^69^ Alternatively, water‐soluble trimethyl chitosan and alginate can be used as replacement for chitosan in sugar vaccine preparation. When these materials were used to package whole inactivated influenza virus and administered as vaccines, high IgG titers were elicited in immunized mice and rabbits.^70^

### 2.2 Synthetic nanoparticles

#### 2.2.1 Biomolecular

##### Liposomes

Liposomes are formed through self‐assembly upon dispersion of certain amphiphilic lipids in aqueous buffer.^71^ These structures can be modified accordingly to achieve desirable features that suit their application purpose, such as achieving particular size and charge to enable entrapment of antigens to be used in vaccines. Liposomes can provide controlled release of antigen, while their plasticity and versatility enable them to overcome biological barriers, such as mucosa and skin. They can gain access to APCs via IM or SC injection routes and be used as both delivery vehicle and immunopotentiator.^72^ Advanced methods to produce liposomal vaccines, including lyophilization, cryoprotection, and sterilization, can enhance chemical stability of the lipids and widen their applicability in vaccine development.^73^

Hong and colleagues reported higher virus‐specific antibodies with long‐lasting protective immunity and 100% survival rate against lethal viral challenges for a cationic liposome‐DNA complex (CLDC)‐adjuvanted influenza vaccine candidate compared to un‐adjuvanted formulation.^74^, ^75^ A similar enhanced immunogenicity effect was reported in a study comparing between vaccination with the commercial FLUZONE® Quadrivalent alone and FLUZONE® Quadrivalent with CLDC. Only the Fluzone/CLDC‐vaccinated animals had lower virus replication when challenged with H1N1 influenza viruses.^76^ In another study, vaccination with liposomes containing HA and NA from various influenza strains and IL‐2/GM‐CSF as an adjuvant (INFLUSOME‐VAC) resulted in increased HAI titers compared to the control groups that received commercial influenza vaccines.^77^

Studies have shown that the dose of liposomes used in vaccines can determine the types of immune response generated. Vaxfectin, which is a commercial liposome‐based adjuvant, induced strong humoral responses in mice when used at a high dose (900 µg) with trivalent influenza vaccine (TIV), while at 30 µg of Vaxfectin, antibody responses were not induced but the amount of interferon‐γ (IFN‐γ) secreting T cells was increased up to 18‐fold.^78^ In agreement with this study, two other cationic lipid‐based adjuvants (DC‐Cholesterol and ceramide carbamoyl‐spermine) were also shown to enhance humoral responses at higher doses and cellular responses at lower doses.^71^, ^79^ This immune‐modulatory property can be explored in influenza vaccine development to produce the required immune responses just by dose modifications.

##### Virosomes

Virosomes are lipid vesicles that incorporate virus‐derived protein and are devoid of viral genome and internal proteins.^80^ The membrane proteins can either be produced via recombinant technology or purified from the corresponding viruses. During surface protein purification, virus membrane is normally solubilized and reconstructed using mild detergents without causing denaturation. After solubilization, nucleocapsid and other viral components will be removed via ultracentrifugation.^81^

Virosomes are biodegradable, non‐toxic and do not induce antiphospholipid antibody responses.^82^ Influenza virus is most commonly used for virosome production, with each virosome averaged at approximately 150 nm in diameter. Almeida et al was the first to generate lipid vesicles containing NA and HA proteins derived from influenza.^80^ Virosomes are better adjuvants compared to liposomes because they can protect pharmaceutically active substances from proteolytic degradation at low pH within the endosomes before reaching the cytoplasm.^83^, ^84^ In addition, virosomes are good adjuvant candidates as they can specifically target APCs and effectively stimulate host B‐ and T‐cell responses against attached antigens, as well as surface HA proteins.^85^ When used at 10‐fold lower dose, a virosome/DNA vaccine complex (consisting of NP‐encoding plasmid attached to influenza virosomes) was reported to induce comparable T‐cell responses in mice that were vaccinated with NP plasmid without virosomes.^86^ Inflexal® V, a trivalent virosome subunit vaccine suitable for use in all age‐groups, is an example of a commercially available virosome‐based influenza vaccine.^87^

##### Immune‐stimulating complexes

Immune‐stimulating complexes (ISCOMs) are particulate adjuvant systems composed of antigen, cholesterol, phospholipid, and saponin.^88^ They are hollow, cage‐like particles of around 40 nm in diameter.^89^ ISCOMs combine the advantages of a particulate carrier system with the presence of an in‐built immunopotentiator (Quil A) and consequently have been found to be more immunogenic than liposomes.^90^ They also required substantially less antigen and other adjuvant to induce immunity in the host than vaccination with simple mixtures of free antigen and saponins.^91^ The use of ISCOM in vaccine formulations needs standardized procedures to produce high‐quality finished vaccines with assured batch‐to‐batch consistency. Heterogeneous mixture of ISCOM components can be separated and purified by reversed phase HPLC to eliminate potential toxic fractions in the vaccine preparation.^92^, ^93^

The use of ISCOM containing influenza viral proteins has been reported to enhance the CD8+ immune responses in mice and humans.^94^, ^95^ Matrix M is a third generation ISCOM and was successfully used as an adjuvant for a H7N9 VLP vaccine in a phase II clinical trial, where the adjuvanted VLP vaccine showed significantly higher seroconversion rates after vaccination compared to non‐adjuvanted VLP vaccine.^47^, ^96^ Also, in a phase I clinical trial involving 60 healthy adults, it was demonstrated that a Matrix M‐adjuvanted H5N1 (NIBRG‐14) vaccine has an acceptable safety profile, capacity for antigen dose sparing and it induced a balanced Th1/Th2 antibody and cellular responses, including multifunctional T cells.^97^ Furthermore, this vaccine elicited protection against highly pathogenic avian influenza A (H5N1) virus challenge in pre‐clinical murine studies.^98^, ^99^

#### 2.2.2 Inorganic NPs

There is now increasing interest in the use of inorganic NPs as adjuvants in vaccine development. An example of inorganic NPs is gold NPs (AuNPs), which have properties that allow conjugation of target antigens or adjuvant at high densities onto their surface.^100^, ^101^ Being a natural element, synthetic AuNPs will not induce carrier‐specific immunity following immunization.^102^ It has been shown that a vaccine candidate made of immobilized M2e on AuNPs and soluble cytosine phosphoguanine‐oligodeoxynucleotides (CpG‐ODN) as an immunopotentiator was able to induce strong M2e‐specific antibody responses and achieve 100% survival rate in mice that were lethally challenged with influenza A/PR/8/34 (H1N1).^103^ The immunogenicity of these NPs can be further enhanced when used together with a bacterial component as immunopotentiator, as shown by Wang et al When both AuNP‐HA (A/Aichi/2/68(H3N2)) and TLR5 agonist flagellin (FliC)‐coupled AuNPs were co‐delivered, stronger cellular immune responses were recorded. In addition, compared with the AuNP‐HA alone group, the addition of AuNPs‐FliC improved mucosal B‐cell responses, as characterized by elevated influenza specific IgA and IgG levels in nasal, tracheal, and lung washes. AuNP‐HA/AuNP‐FliC also stimulated antigen‐specific IFN‐γ secreting CD4+ cell proliferation.^104^

#### 2.2.3 Polymer NPs

Synthetic polymers have unique characteristics such as biocompatibility and versatility due to their chemical structure.^105^ They can be modified in terms of size, surface properties, and composition, which results in a controlled release and protection of drugs. PLGA (poly D,L‐lactide‐co‐glycolide) is a FDA‐approved, biodegradable synthetic polymer used for drug delivery in humans.^106^ They are tuneable and flexible, and their outer surface can be modified to incorporate other polymers such as chitosan for more effective mucosal vaccine delivery.^107^, ^108^ When encapsulated in PLGA NPs, antigens can be prevented from degradation for over four weeks under physiological conditions. Moreover, they can promote antigen internalization by APCs.^109^ PLGA NP‐based vaccines have been reported to improve the immunogenicity of several conventional and recombinant vaccines targeting human and veterinary pathogens.^110^, ^111^ Molecules that can target mucosal APCs can be covalently attached to PLGA NPs for the induction of long‐lasting and potent immune responses.^11^

## 3 CONCLUSION

There is great potential in the use of NPs in influenza vaccine development as they can be used to deliver antigens to target cells, improve antigen stability, promote slow release of antigens, and increase immunogenicity. The availability of recombinant technology allows these nanomaterials to be further modified to achieve and boost the desired immune responses. For example, additional molecules such as TLR ligands can be added to the NPs to allow better stimulation and activation of antigen‐presenting cells. NP‐based vaccines are also safer compared to live attenuated vaccines, which pose a risk to the elderly and immunosuppressed individuals.

While there are clear advantages in using NPs as vaccine carrier and adjuvant, it is not known if results from pre‐clinical studies will translate into success in human clinical trials. To maximize the chance of success, the design of these new generation NP‐based vaccines needs to be guided by comprehensive scientific knowledge on their mechanisms of action. More studies are needed to investigate the specific ways by which different NPs interact with immune cell populations that are involved in antibody production and memory generation. The downstream immune responses such as cytokine production and complement activation should also be characterized in detail, as these responses can be protective but pathological when in excess. In addition, it should be explored if different routes of immunization can impact on the generation of long‐term immunity induced by these NP‐based vaccines. Future studies can also investigate the potential use of several types of NPs in one vaccine formulation to enhance immunogenicity. By enhancing our understanding on these issues, a safer, highly immunogenic and affordable influenza vaccine can be expected in the near future.

## AUTHOR CONTRIBUTIONS

AK‐H involved in writing, reviewing, and editing processes. CLL, PS, and KW involved in supervision, editing, and review process.
